# Optimal cut points of *N*-terminal of the prohormone brain natriuretic peptide (NT-proBNP) in patients with COVID-19

**DOI:** 10.1186/s43044-022-00253-1

**Published:** 2022-03-16

**Authors:** Mohammad Chehrazi, Hanieh Yavarpour, Farzad Jalali, Mehrdad Saravi, Iraj Jafaripour, Mohammad Taghi Hedayati, Kamyar Amin, Roghayeh Pourkia, Saied Abroutan, Mostafa Javanian, Soheil Ebrahimpour, Naghmeh Ziaie

**Affiliations:** 1 Department of Biostatistics and Epidemiology, School of Public Health, Babol University of Medical Sciences, Babol, 4717647745 Islamic Republic of Iran; 2 Department of Cardiology, School of Medicine, Rouhani Hospital, Babol University of Medical Sciences, Ganjafrouz St, Babol, Islamic Republic of Iran; 3 Infectious Diseases and Tropical Medicine Research Center, Health Research Institute, Babol University of Medical Sciences, Babol, Islamic Republic of Iran

**Keywords:** NT-proBNP, COVID-19, Survival time, In-hospital death

## Abstract

**Background:**

COVID19 patients may suffer from multiple cardiovascular complications. Recently, *N*-terminal of the prohormone brain natriuretic peptide (NT-proBNP) was a potentially independent risk factor for COVID-19 in-hospital death. The present study aimed to find new optimal cut points for NT-proBNP across censored survival failure time outcomes in hospitalized COVID-19 patients.

**Results:**

This cohort study was conducted on 272 patients with COVID-19 whose initial records were recorded from March 2020 to July 2020. Demographic characteristics, clinical examinations, and laboratory measurements were collected at the beginning of the admission registered in the patient record system located in the hospital. We used the maximally selected rank statistics to determine the optimal cut points for NT-proBNP (the most significant split based on the standardized log-rank test). Survival time was defined as the days from hospital admission to discharge day. In this cohort study, two optimal cut points for NT-proBNP were 331 (pg/mL) and 11,126 (pg/mL) based on a survival model. The adjusted HR of NT-proBNP for in-hospital death was 3.41 (95% CI: 1.22–9.51, *P* = 0.02) for medium against low category, and 3.84 (95% CI: 1.30–11.57, *P* = 0.01) for high in comparison with low group.

**Conclusions:**

We reported a dramatically increased concentration of NT-proBNP among COVID-19 patients without heart failure in both severe and non-severe cases. Moreover, our study showed that a high level of NT-proBNP was highly associated with the prolonged survival time of patients with COVID-19. NT-proBNP is a strong prognostic indicator of in-hospital death in the second week of admission.

## Background

Severe acute respiratory syndrome coronavirus 2 (SARS-CoV-2) was first found in Wuhan, Hubei Province, China, on 31 December 2019. This virus caused novel coronavirus disease 2019 (COVID-19) [1]. Myocardial injury is a common complication among patients with severe acute respiratory syndrome coronavirus 2 (SARS-CoV-2) infections, correlated with poor outcomes [2]. Different cardiac biomarkers, including cardiac troponin I (cTnI), alpha-hydroxybutyrate dehydrogenase (α-HBDH), myoglobin (Mb), lactate dehydrogenase (LDH), creatine phosphokinase (CPK), creatinine phosphokinase-muscle/brain (CPK-MB), aspartate aminotransferase (AST), and brain natriuretic peptide (BNP)/*N*-terminal of the prohormone brain natriuretic peptide (NT-proBNP), increase to a different extent among patients with Coronavirus disease of 2019 (COVID-19). Although these biomarkers are increased during cardiac injury, not all are specific to myocardial damage. However, cTnI, CPK-MB, and NT-proBNP/BNP are cardiac biomarkers, specifically showing myocardial injury, and are reported to increase, especially in severe COVID-19 patients in the Intensive Care Unit (ICU) [3]. Cardiac involvement consists of different presentations such as arrhythmia, myocarditis, cardiogenic shock, acute myocardial injury, and heart failure with variable severity [4]. In another study on 138 patients with COVID-19, 7.2% and 16.7% of the subjects suffered from acute cardiac injury and arrhythmia, respectively. This fraction increases, respectively, to 22.2% and 44% in patients with severe conditions [3]. The main underlying mechanism of SARS-CoV-2-induced cardiac damage is not fully identified; however, some evidence suggests different pathogenic pathways, leading to myocardial injury in SARS-CoV-2 infection as follows: (a) Direct viral infection through intracellular replication leading to cardiomyocyte degeneration and necrosis can result in loss of cardiac function and arrhythmia [5]. (b) Moreover, the virus may exert its impact through binding to its specific receptor Angiotensin-Converting Enzyme 2 (ACE2), which is highly expressed in the heart, as well as the lungs [6, 7]. (c) Immune-related pathway is the other underlying mechanism for SARS-CoV-2 myocardial injury [8, 9]. Several lines of evidence reported high amounts of inflammatory markers in COVID-19 patients. An essential feature regarding these pathways is investigating prognostic cardiac biomarkers, reflecting the abovementioned processes. BNP and NT-proBNP are released from cardiac myocytes in response to increases in wall stress [10], providing robust and independent prognostic value in patients with various cardiovascular diseases such as heart failure and acute coronary syndromes valvular aortic stenosis, and stable coronary artery disease [11]. Different studies showed increased levels of NT-proBNP in COVID-19 patients [2, 12]. Recently Gao et al. demonstrated that higher levels of NT-proBNP are associated with an increased risk of mortality in patients with severe COVID-19 [12]. Although the pathophysiological pathway behind up-regulation of NT-proBNP in these patients is not fully elucidated, some suggested mechanisms exist. Acute respiratory distress syndrome induces right heart strain [13], inflammation [14], ischemia [15], and hypoxemia [16] that are stimulated by SARS-CoV-2 direct/indirect injury. These suggested underlying pathways result in increased ventricular wall stress and subsequent release of NT-proBNP. However, some mentioned mechanisms are reported to increase NT-proBNP levels, independent of increased heart wall stress [17].

According to Gou et al., NT-proBNP levels increased dramatically in those who died during hospitalization, but no such dynamic changes in NT-proBNP levels were observed in survivors [18]. As a result, we designed this study to find new optimal cut points in a larger population of COVID-19 patients, investigate the prognostic value of NT-proBNP in predicting survival time, and collect data on the time-dependent predictive accuracy of NT-proBNP levels.

## Methods

### Study design and population

This is a historical cohort study of all COVID-19 patients whose initial records were reported from March 2020 to July 2020. According to the following criteria, patients were divided into two groups: severe and non-severe: (1) rest oxyhemoglobin saturation (SpO_2_) less than 93%, or (2) oxygenation index (arterial oxygen tension/inspired oxygen fraction, PaO_2_/FiO_2_) less than 300 mmHg, or (3) respiratory rate greater than 30/min. Patients with a history of heart failure and a lack of laboratory measurements were excluded from the study. Written informed consent was obtained from all patients before the study. The Institutional Review Boards approved the study of the participating institutions, Rouhani Hospital and Babol University of Medical Sciences, and conducted by the guideline of the University Ethics Committee, approval No 724133037.

### Initial records and follow-up time

Demographic characteristics, clinical examinations, and laboratory measurements were collected at the beginning of the registered admission in the patient record system located at the hospital. All laboratory measurements were carried out with the same standard and kits at the same laboratory. Survival time was counted from hospital admission to death related to COVID-19 when COVID-19 was the underlying cause of death. Follow-up time was censored when a patient either died of an underlying cause, except for COVID-19, or was still alive upon discharge.

### Statistical analysis

We used the maximally selected rank statistics to determine the optimal cut points for NT-proBNP (the most significant split based on the standardized log-rank test). This outcome-oriented method provides a cut point value that corresponds to the most significant relationship with the outcome (here, survival time to in-hospital death). Classification of the population into three groups based on survival time was carried out in the application *Evaluate Cutpoints,* using the hierarchical clustering method (function *rhier* from the *Rolr* package). *Evaluate Cutpoints* is an application developed using the R language [19], Shiny framework, and R packages (R version 3.4.1), including *survival*, *survMisc*, *OptimalCutpoints* [20], *maxstat* [21], *Rolr*, *ggplot2*, *GGally,* and *plotly*. Firstly, the algorithm splits the cohort into two groups by estimating the optimal cut point with the highest log-rank statistics. The procedure is then repeated in the resulting groups to obtain two supplementary cut-off values. The second optimal cut point is the one with larger test statistics.

The application omits all rows (observations) with NA values. After the optimal cut points were determined, the Kaplan–Meier estimation method with log-rank test was used to estimate cumulative survival curves of in-hospital death. To estimate the impact of the prognostic factors, including NT-proBNP, with new categories and the other covariates on survival time, the Cox proportional hazard model was used for censored survival data. Risk estimates are presented as hazard ratios (HRs) with 95% CI. The assumption of proportional hazards was met based on Schoenfeld residual analysis. No multicollinearity between the independent variables (tested by variance inflation factor analysis) was found. Analysis of variance (ANOVA), Bonferroni as a post hoc test, and the Chi-square test were used to compare continuous and categorical variables among NT-proBNP (pg/mL) categories. All p values were two-tailed, and p < 0.05 was significant.

## Results

### Patient characteristics

**Fig. 1:**
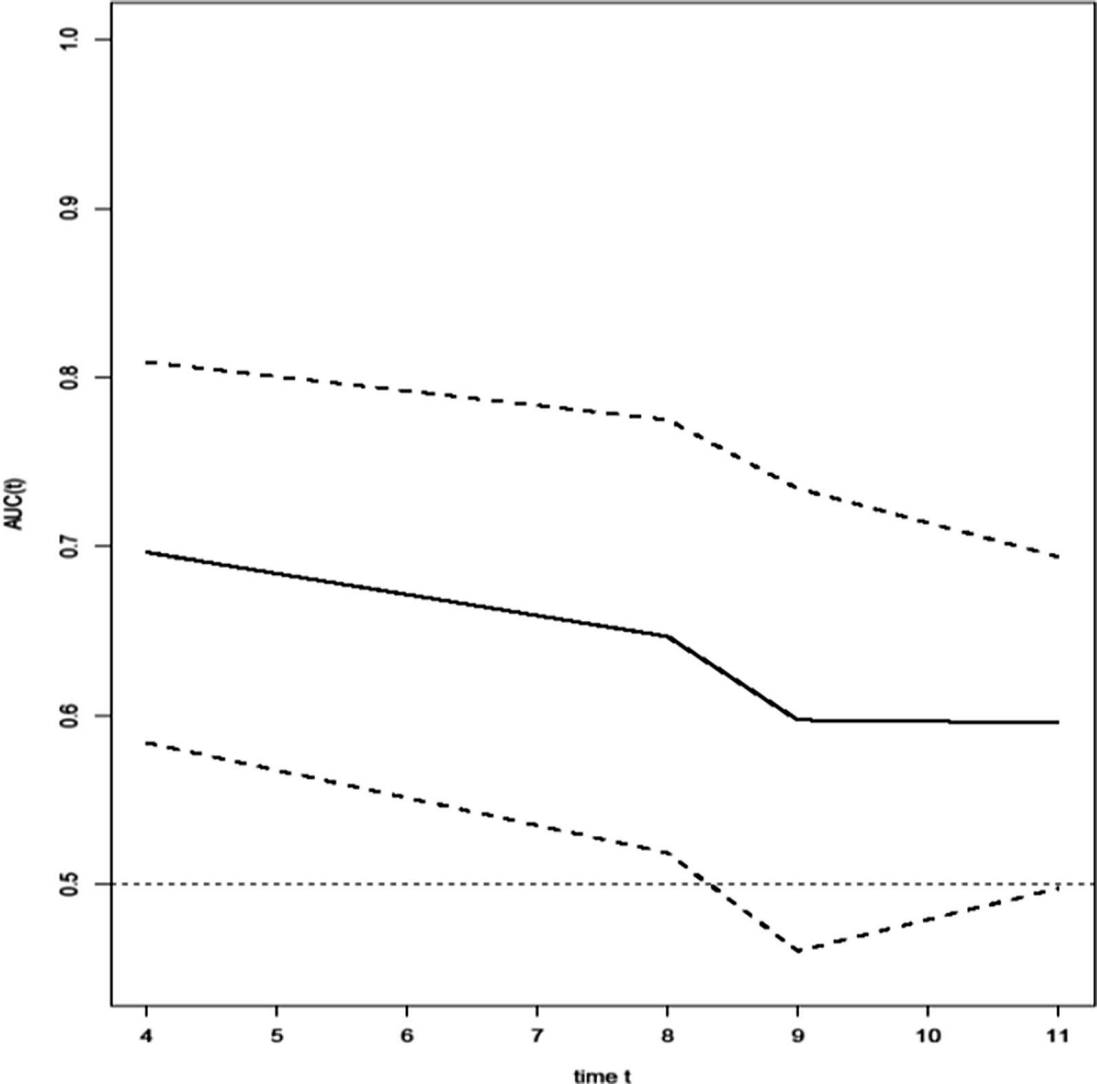
Inverse probability of censoring weighting estimation of cumulative/dynamic time-dependent ROC curve for NT-proBNP patients with COVID-19

The essential characteristics of the 272 participants were divided into three groups: low (NT-proBNP ≤ 311 pg/mL), medium (311 pg/mL < NT-proBNP ≤ 11,126 pg/mL), and high NT-proBNP (NT-proBNP > 11,126 pg/mL, Table 1), based on the cut-off value determined by inverse probability of censoring weighted (IPCW) estimation of dynamic time-dependent receiver operating characteristic (ROC) curve, which considers outcome (in-hospital death), as a time-dependent variable (Fig. 1). This graph depicted the area under the curve (AUC) as a function of follow-up time. The discrimination ability of NT-proBNP decreased until day 9, at which it became constant. Patients in the low NT-proBNP group were significantly younger with a lower prevalence of hypertension (HTN), coronary artery disease (CAD), diabetes mellitus, and kidney disease, as well as lower blood urea (BUN), procalcitonin (PCT), creatinine, white blood cell (WBC) and a lower level of troponin than those in the medium and high NT-proBNP groups (*P* < 0.0001). The odds of a COVID-19 patient having a positive troponin were zero for a low NT-proBNP patient but 5 times higher for a high NT-proBNP patient. The odds of severe status were statistically higher in those with high levels of NT-proBNP than in those with low levels (*P* = 0.004). Furthermore, patients with high NT-proBNP levels were more likely to be admitted to the ICU (*P* = 0.008). Other characteristics, such as gender, temperature, level of C-reactive protein (CRP), history of respiratory disease (odds of having respiratory disease among Low NT-proBNP: 0.065; Medium: 0.072; High: 0.045), and cancer (odds of having cancer among Low NT-proBNP: 0.088; Medium: 0.047; High: 0.045) showed no significant difference between the three groups with different levels of NT-proBNP (Table 1).

**Table 1:** Baseline measurements of included patients with COVID-19 according to level of NT-proBNP

Measures	Total	Low NT-proBNP ≤ 331	Medium NT-331 < NT-proBNP ≤ 11,126	High NT-proBNP > 11,126	*P* value
Age (year)	61.69 (17.41)	53.68 (17.45)	64.06^a^ (16.22)	61.31 (19.53)	0.001
Gender (F/M)	127/145	22/27	82/95	23/23	0.87
ICU admission (yes/no)	138/134	18/31	89/88	31/15^a,b^	0.01
severity (severe/non-severe)	168/104	22/27	110/67^a^	36/10^a,b^	0.004
Hospital death (yes/no)	80/188	4/44	54/121^a^	22/23^a,b^	< 0.0001
History of HT (yes/no)	136/136	18/31	91/86	27/19	0.08
History of CAD (yes/no)	60/212	7/42	37/140	16/30	0.045
History of DM (yes/no)	72/200	12/37	48/129	12/34	0.93
History of resp (yes/no)	17/255	3/46	12/165	2/44	0.83
History of cancer (yes/no)	14/258	4/45	8/169	2/44	0.57
History of KD (yes/no)	16/256	2/47	11/166	3/43	0.84
History of ARB yes/no	112/160	16/33	74/103	22/24	0.31
WBC (10^3^/µL)	10.38 (5.61)	8.46 (4.8)	10.61 (5.99)	11.55^a^ (4.34)	0.02
CRP (mg/L)	100.61 (115.22)	74.89 (69.57)	108.16 (132.30)	97.47 (65.88)	0.21
BUN (mg/dL)	34.85 (30.55)	23.72 (25.03)	34.30 (27.20)	50.46^a,b^ (42.17)	0.0001
PCT (ng/mL)	3.28 (11.28)	0.90 (2.54)	2.85 (10.42)	6.79 (16.81)	0.09
Creatinine (mg/dL)	1.57 (1.47)	1.11 (1.10)	1.50 (1.14)	2.42^a,b^ (2.43)	0.0001
Troponin (positive/negative)	64/205	0/49	25/105^a^	39/7^a,b^	< 0.0001
NT-proBNP (pg/mL)	6362.17 (9013.81)	131.02 (113.99)	3516.54^a^ (2838.85)	23,949.17^a,b^ (8213.34)	< 0.0001

*HT* Hypertension, *CAD* Coronary Artery Disease, *DM* Diabetes Mellitus, *Resp* Respiratory Disease, *KD* kidney Disease, *ARB* Angiotensin II Receptor Blockers, *WBC* White Blood Cell, *CRP* C-reactive protein, *BUN* Blood Urea Nitrogen, *PCT* Procalcitonin, *NT-proBNP* N-terminal pro-brain natriuretic peptide

^a^Significant in comparison with low

^b^Significant in comparison with medium

### Dynamic time-dependent ROC curve and the cut points

**Fig. 2:**
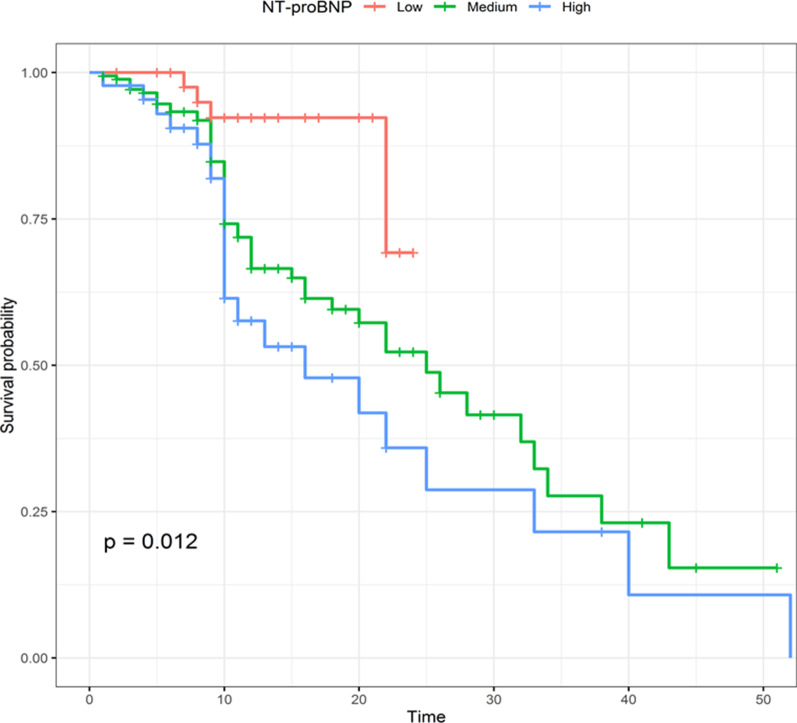
Kaplan–Meier curve of the cumulative survival rate of patients with COVID-19 categorized into three groups based on NT-proBNP cut points. Red line is for NT-proBNP ≤ 331; green line is for 331 < NT-proBNP ≤ 11,121; Blue line is for NT-proBNP > 11,121

Using the hierarchical clustering method, we stratified the sample of patients into three groups with simultaneous consideration of the survival time. The first optimal cut point for NT-proBNP was determined to be 331 pg/mL and the second was 11,126 pg/mL. Table 2 shows the diagnostic accuracy indices and AUC for two cut points. The longer the time, the lower was the AUC. AUC was 69.64% on day 4 and 59.57 on day 11. During the follow-up period, sensitivity and negative predictive value (NPV) were both excellent. On day 4, sensitivity and NPV were both 100% for the first cut point of 331 ng/mL. Specificity and positive predictive value (PPV), on the other hand, appeared to perform better over time. Follow-up time ranged from 1 to 52 days (median, 9 days). Before discharge from the hospital, 107 patients with COVID-19 (31.20%) died. Overall, 10-, 22-, and 38-day survival rates were 75%, 50%, and 25%, respectively. Kaplan–Meier plots were generated and showed statistically significant differences in survival days between patients with low, medium, and high levels of NT-proBNP (Fig. 2). Moreover, a low level of NT-proBNP was correlated with the most favorable prognosis, while high levels were associated with the worst prognosis (IR = 0.007 for low, = 0.025 for medium, and = 0.037 for high, log-rank test *P* = 0.012) (Table 3). Furthermore, the estimated median survival time was lower for high level (*t* = 16) of NT-proBNP than the two other groups (Low-level *t* = not available; medium-level *t* = 25).

**Table 2:** Predictive accuracy measures at cut point = 331 estimated using inverse probability of censoring weighting (IPCW)

	Day 4	Day 8	Day 9	Day 11
Survivor	239	183	136	83
Death	6	16	20	49
Censored	23	69	112	136
SN	100 (SE = 0)	92.94 (SE = 6.79)	88.84 (SE = 7.41)	94.30 (SE = 3.21)
SP	19.67 (SE = 2.58)	19.67 (SE = 2.94)	17.65 (SE = 3.28)	18.07 (SE = 4.23)
PPV	2.81 (SE = 1.13)	7.44 (SE = 1.86)	9.42 (SE = 2.14)	28.36 (SE = 3.77)
NPV	100 (SE = 0)	97.57 (SE = 2.41)	94.25 (SE = 3.99)	90.22 (SE = 5.56)
AUC	69.64 (SE = 5.75)	64.68 (SE = 6.54)	59.73 (SE = 6.98)	59.57 (SE = 5.01)

**Table 3:** Predictive accuracy measures at cut point = 11,126 estimated using IPCW

	Day 4	Day 8	Day 9	Day 11
Survivor	239	183	136	83
Death	6	16	20	49
Censored	23	69	112	136
SN	16.56 (SE = 15.17)	24.97 (SE = 10.85)	24.98 (SE = 9.73)	27.40 (SE = 6.55)
SP	83.68 (SE = 2.39)	83.61 (SE = 2.74)	82.35 (SE = 3.28)	83.13 (SE = 4.12)
PPV	2.30 (2.28)	9.57 (SE = 4.61)	12.01 (SE = 5.20)	35.84 (SE = 8.89)
NPV	97.74 (SE = 1.00)	94.13 (SE = 1.65)	91.92 (SE = 2.02)	76.90 (SE = 3.55)

### Cox proportional hazard model

The results of the multivariable Cox proportional hazard model are given in Table 4. This model evaluated the possible effect of proBNP on survival time of the patients with COVID-19 in days until death, adjusted for the potential confounding effects of the covariates given in Table 1. After adjusting for the covariates, the adjusted HR of NT-proBNP for in-hospital death was 3.41 (95% CI: 1.22–9.51, *P* = 0.02) for medium against low category, and 3.84 (95% CI: 1.30–11.57, *P* = 0.01) for high, in comparison with the low group. In addition, the adjusted HRs of gender, illness status, and PCT also showed a significant prognostic impact on the hazard of in-hospital death. Male patients were more likely to die than females with adjusted HR = 2.10 (95% CI: 1.31–3.36, *P* = 0.002). Severe COVID-19 patients died at a higher rate than non-severe patients, with an adjusted HR of 3.32 (95% CI: 1.59–6.97, *P* = 0.001).

**Table 4:** Summary of survival time and incidence rate of patience with COVID-19 for three categories of NT-proBNP

NT-proBNP	Time at risk	IR (95% CI)	Failure time		
			25%	50%	75%
Low (*n* = 49)	538	0.007	22	–	–
Medium (*n* = 177)	2111	0.025	10	25	38
High (*n* = 46)	584	0.037	10	16	33

*IR* incidence rate

## Discussion

Some interesting and novel aspects of our study have been emphasized here. First, this is the first study regarding the informative value of NT-proBNP levels to predict a patient’s survival time, which is defined as the time from admission to death due to SARS-CoV-2 infections. According to our results, this correlation became significant even after adjustment for different confounding factors, as shown in Table 1. We demonstrated the statistical correlation by suggesting two lower and higher cut points. In this line, NT-proBNP levels lower than 331 pg/mL and higher than 11,126 pg/mL were associated with the longest and the shortest duration from admission to death, respectively. Second, we indicated for the first time that the predictive value of the NT-proBNP for COVID-19 patients’ survival time varied during times of follow-up. Third, in contrast to previous studies, we did not include only severely affected patients, but we enrolled both severely affected and non-severe patients, and our results and two suggested cut-off values are accounted for both groups. Hence, interestingly, not only severely affected patients, but also non-severe ones with NT-proBNP higher than 11,126 pg/mL had the shortest survival time (Table 5).

**Table 5:** Adjusted effect of NT-proBNP on in-hospital death of patients with COVID-19 estimated through Cox proportional–hazard regression model

Model	Adjusted HR	95% CI	*P* value
NT-proBNP			
Low	Reference		
Medium	3.41	1.22–9.51	0.02
High	3.88	1.30–11.57	0.01
Sex			
Female	Reference		
Male	3.28	1.75–6.12	< 0.001
Age	1.01	0.99–1.02	0.26
ICU Admission			
No			
Yes	3.05	1.49–6.27	0.002
History of CAD			
No	Reference		
Yes	0.79	0.38–1.66	0.54
History of HT			
No	Reference		
Yes	0.37	0.14–1.02	0.055
History of cancer			
No	Reference		
Yes	0.96	0.40–2.99	0.93
History of KD			
No	Reference		
Yes	1.06	0.38–2.96	0.91
History of Resp			
No	Reference		
Yes	0.29	0.07–1.23	0.09
History of DM			
No	Reference		
Yes	0.73	0.43–1.23	0.24
Severity			
Non-severe	Reference		
Severe	3.32	1.59–6.97	0.001
NT-proBNP*severity	0.7	0.19–2.52	0.58
WBC (10^3^/µL)	1.01	0.99–1.00	0.6
BUN (mg/dL)	1	0.99–1.01	0.0.22
CRP (mg/L)	1	0.84–1.19	0.99
PCT (ng/mL)	1.02	1.01–1.04	0.002
Creatinine (mg/dL)	0.99	0.84–1.18	0.96
Troponin			
Negative			
Positive	1.02	0.56–1.85	0.95

Based on several lines of evidence, myocardial injury is a common complication among hospitalized COVID-19 patients [2, 12]. Moreover, SARS-CoV-2 infection is accompanied by more complications in patients with cardiac injury than those without [22]. Accordingly, laboratory cardiac biomarkers in these patients are changing to varying degrees and are evidenced to predict the risk of worsening prognosis and in-hospital death in COVID-19 patients, both with and without myocardial injury [23]. To date, different studies explained the association of higher NT-proBNP levels with a higher mortality rate [12], severe illness status [22], higher levels of other cardiac markers [2, 22] among patients with SARS-CoV-2 infection. However, none of them evaluated this cardiac biomarker's informative value to estimate a patient’s survival time. Among these studies, Gao et al*.* suggested an NT-proBNP level of 88.64 pg/mL as the best cut-off value for predicting a patient’s mortality rate [12]. This cut point, however, was far lower than both our low (331 pg/mL) and high (11,126 pg/mL) cut-off values. Moreover, this difference was consistent between the average level of NT-proBNP reported in their study (137.30 pg/mL) and our study (6362.17 pg/mL). This may be explained by their small study population (n = 54 vs. n = 272 in our study) as in another study, among a large cohort of COVID-19 patients, the medium of NT-proBNP was reported as 847.5 pg/mL with the median peak of 1047.0 pg/mL [2].

One surprising finding was the time dependency of NT-proBNP to predict a patient’s survival time. Using a time-dependent dynamic ROC curve enabled us to estimate a patient’s survival time in different time courses. Interestingly, we observed that NT-proBNP predictive information rose during the follow-up time. Furthermore, our lower cut point (331 pg/mL) had a higher negative predictive value (NPV) rather than our higher cut point (1126 pg/mL) regarding the estimate of durations from admission to death. This difference in prognostic value can, in part, be ascribed to the time-dependent release of NT-proBNP. Little data are available on the time course of NT-proBNP levels concerning the onset of symptoms. Weber et al. studied the dependency of NT-proBNP values on the time interval from the beginning of symptoms. As they reported, the highest value was measured 24–36 h after the start of symptoms [11]. Our study observed that NT-proBNP acts as a more sensitive prognostic biomarker during post-admission. Hence, further studies are advised for serial sampling from patients with COVID-19 to measure the dependency of NT-proBNP values on admission duration and find the highest predictive value of this biomarker.

Calvo-Ferna´ndez et al*.,* in a single-center cohort study, evaluated the association between mortality rate and cardiac injury in a total of 416 hospitalized COVID-19 patients [24]. Interestingly, they found that patients with cardiac injury had a shorter duration from both symptom onsets/admission to follow-up than patients without cardiac injury. Moreover, patients with more severe acute illness are indicated by abnormal laboratory markers such as higher concentrations of NT-proBNP, creatinine, high-sensitivity (hs)-TNI, and PCT. Consistent with previously published articles in our study, patients with higher levels of NT-proBNP and higher death likelihoods were at a higher risk of HTN, CAD, diabetes mellitus, kidney disease and had higher levels of BUN creatinine, leucocytes, and PCT. Although both PCT and CRP are inflammatory parameters, different time takes to reach the peak value. Up-regulation of PCT in systemic inflammation or infection happens within 2−4 h, comes peak values in 8−24 h, and remains for as long as the inflammation processes. The half-life of PCT is about 24 h.

By comparison, CRP takes 12−24 h to reach the peak and persists for up to 3–7 days. Therefore, PCT values increase earlier and get the normal range more rapidly than CRP [25]. This makes PCT a potential marker to diagnose the disease in its earlier stage, and better monitor its progression. Consistent with this report, in the study of Caro-Codón et al., inflammatory markers including CRP did not significantly correlate with the first NT-proBNP determined at admission time; however, this relation became significant at the time of NT-proBNP peak measurement [2].

In agreement with previous articles, the number of troponin-positive patients was significantly higher in our high category of NT-proBNP and associated with a shorter duration from admission to death. Currently, Calvo-Ferna´ndez et al*.* revealed that NT-proBNP improved the accuracy of high-sensitivity cardiac troponin T (hs-cTnT), as a prognostic factor of death and the analyzed outcomes [25]. Moreover, troponin is a marker of myocardial necrosis [26]. Hence, this relationship between NT-proBNP and troponin among patients without current or previous history of heart failure (excluded from this study) may suggest myocardial necrosis as possible stimuli for NT-proBNP elevation, as well as troponin. Of note, it is not clear what mechanisms exactly underlie NT-proBNP elevation; however, different possible pathophysiologic causes such as hemodynamic deterioration, myocardial ischemia, derangements in volume loading conditions, and hypoxia are suggested as stimulators [27, 28].

Inflammation by itself is suggested as a possible driver for a higher level of circulating natriuretic peptides [14]. Studies of blood assessment among patients with COVID-19 indicated a large number of inflammatory cytokines [29]. The plasma of newly diagnosed COVID-19 patients contains different inflammatory cytokines such as interleukins IL-1β, IL-1RA, IL-7, tumor necrosis factor-α (TNF-α), platelet-derived growth factor (PDGF), and vascular endothelial growth factor (VEGF) [7] as well as inflammatory markers that are higher in severe COVID-19 patients rather than non-severe patients [30]. In this regard, we examined if the correlation of NT-proBNP levels with patients’ survival time changes in severe versus non-severe patients. Interestingly, this correlation remained the same in both groups. We also investigated our three low, medium, and high patient groups for their admission to the ICU. As shown, patients in the high group (NT-proBNP > 1126 pg/mL) were more likely to be admitted to ICU rather than medium and low groups. Huang et al. demonstrated that ICU patients with COVID-19 had higher plasma levels of inflammatory markers compared with the non-ICU COVID-19 patients [9]. Hence, there is a possible stimulatory role of inflammation on NT-proBNP release.

Hypoxia is the other possible mechanism besides NT-proBNP release [16]. Li et al. mentioned that pneumonia induced by SARS-CoV-2 infection results in critical gas exchange obstruction, causing hypoxemia. This hypoxic state decreases the energy supply by cell metabolism, which results in increasing anaerobic fermentation, intracellular acidosis, and oxygen-free radical formation, and finally damaging the phospholipid layer of the cell membrane [4]. To investigate whether hypoxia induced the NT-proBNP up-regulation, we evaluated the patient’s SpO_2_ saturation at admission time and NT-proBNP concentrations. Although we did not find a significant correlation, serial measuring of O_2_ saturation was needed to verify this association.

Moreover, regulation of NT-proBNP level through gene expression suggests a possible correlation between BNP genotype polymorphism and extraordinary up-regulation of NT-pro BNP. The high level of NT-pro BNP in the high category (> 11,126 pg/mL) raises the question of whether any polymorphism is responsible for highly increased NT-proBNP. This hypothesis was supported by some evidence from different studies, which indicated that some polymorphisms in the BNP gene are associated with a significantly high NT-proBNP level. For instance, some researchers have shown that rs198389 polymorphism is associated with a higher level of NT-proBNP and BNP [31, 32]. However, multicenter studies are needed to shed light on this possible relationship.

## Conclusions

To our knowledge, this is the first report regarding the informative value of NT-proBNP concerning COVID-19 patient’s times from hospital admission to death. In the present study, we reported a dramatically increased concentration of NT-proBNP among COVID-19 patients without heart failure in both severe and non-severe cases. We also, for the first time, suggested two optimal cut-off values, predicting a patient’s survival time and prognosis. Our study presented novel data, which can guide clinicians to better manage patients with COVID-19, based on the NT-proBNP plasma level at the hospital admission time. We demonstrated the time-dependent accuracy of NT-proBNP measurement. Our study showed that a high level of NT-proBNP was highly associated with the survival time of patients (in-hospital death) with COVID-19. NT-proBNP is a strong prognostic indicator of in-hospital death in the second week of admission. More studies with follow-up measurements of NT-proBNP would be warranted. Furthermore, we observed that NT-proBNP predictive value increased during follow-up, but there may be a time peak for this biomarker with the highest predictive accuracy. There is no data regarding this, and we recommend further study to assess if serial sampling of NT-proBNP is a better investigator of disease survival time and mortality rate.

## Data Availability

The data used to support the findings of this study are included within the article.

## Abbreviations

ARB: Angiotensin II receptor blockers
AUC: Area under curve
BUN: Blood urea nitrogen
CAD: Coronary artery disease
COVID-19: Coronavirus disease 2019
CPK: Creatinine phosphokinase
CRP: C-reactive protein
DM: Diabetes mellitus
HT: Hypertension
KD: Kidney disease
NPV: Negative predictive value
NT-proBNP: *N*-terminal of the prohormone brain natriuretic peptide
PPV: Positive predictive value
PCT: Procalcitonin
Resp: Respiratory disease
WBC: White blood cell
